# Peripheral quantitative computed tomography in the assessment of bone mineral density in anti-TNF-treated rheumatoid arthritis and ankylosing spondylitis patients

**DOI:** 10.1186/s12891-021-04708-5

**Published:** 2021-09-23

**Authors:** Balázs Juhász, Katalin Gulyás, Ágnes Horváth, Edit Végh, Anita Pusztai, Ágnes Szentpétery, Zsófia Pethő, Nóra Bodnár, Attila Hamar, Levente Bodoki, Harjit Pal Bhattoa, Éva Szekanecz, Katalin Hodosi, Andrea Domján, Szilvia Szamosi, Csaba Horváth, Sándor Szántó, Gabriella Szűcs, Hennie G. Raterman, Willem F. Lems, Oliver FitzGerald, Zoltán Szekanecz

**Affiliations:** 1 Department of Oncology, Faculty of Medicine, University of Debrecen, Debrecen, Hungary; 2 Division of Rheumatology, Department of Internal Medicine, Faculty of Medicine, University of Debrecen, Nagyerdei str. 98, Debrecen, 4032 Hungary; 3 Department of Rheumatology, Uppsala University Hospital, Uppsala, Sweden; 4 Conway Institute for Biomolecular Research, University College Dublin, Dublin, Ireland; 5 Department of Laboratory Medicine, Faculty of Medicine, University of Debrecen, Debrecen, Hungary; 6 First Department of Medicine, Semmelweis University, Budapest, Hungary; 7 Department of Sports Medicine, Faculty of Medicine, University of Debrecen, Debrecen, Hungary; 8 Department of Rheumatology, Northwest Clinics, Alkmaar, The Netherlands; 9 Amsterdam Rheumatology and Immunology Centre, Amsterdam, The Netherlands

**Keywords:** Rheumatoid arthritis, Ankylosing spondylitis, Osteoporosis, Bone density, Peripheral quantitative computed tomography, Biologics

## Abstract

**Abstract:**

**Introduction:**

Rheumatoid arthritis (RA) and ankylosing spondylitis (AS) are associated with osteoporosis. There have not been many peripheral quantitative computed tomography (QCT) studies in patients receiving biologics. We assessed volumetric and areal bone mineral density (BMD) by forearm QCT and dual-energy X-ray absorptiometry (DXA), respectively in addition to laboratory biomarkers in these arthritides.

**Methods:**

Forty RA and AS patients treated with either etanercept (ETN) or certolizumab pegol (CZP) were undergoing follow-ups for one year. Volumetric and areal BMD, as well as parathyroid hormone (PTH), osteocalcin, RANKL, 25-hydroxyvitamin D (VITD), P1NP, CTX, sclerostin (SOST), Dickkopf 1 (DKK-1) and cathepsin K (CATHK) were determined.

**Results:**

We did not observe any further bone loss during the 12-month treatment period. Volumetric and areal BMD showed significant correlations with each other (p<0.017 after Bonferroni’s correction). Trabecular QCT BMD at baseline (p=0.015) and cortical QCT BMD after 12 months (p=0.005) were inversely determined by disease activity at baseline in the full cohort. Trabecular QCT BMD at baseline also correlated with CTX (p=0.011). In RA, CRP negatively (p=0.014), while SOST positively (p=0.013) correlated with different QCT parameters. In AS, RANKL at baseline (p=0.014) and after 12 months (p=0.007) correlated with cortical QCT BMD. In the full cohort, 12-month change in QTRABBMD was related to TNF inhibition together with elevated VITD-0 levels (p=0.031). Treatment and lower CATHK correlated with QCORTBMD changes (p=0.006). In RA, TNF inhibition together with VITD-0 (p<0.01) or CATHK-0 (p=0.002), while in AS, treatment and RANKL-0 (p<0.05) determined one-year changes in QCT BMD.

**Conclusions:**

BMD as determined by QCT did not change over one year of anti-TNF treatment. Disease activity, CATHK, RANKL and VITD may be associated with the effects of anti-TNF treatment on QCT BMD changes. RA and AS may differ in this respect.

**Supplementary Information:**

The online version contains supplementary material available at 10.1186/s12891-021-04708-5.

## Key points

- Peripheral quantitative CT is suitable to assess volumetric bone mineral density in RA and AS patients
- In this study, BMD did not change over one year.
- Some bone biomarkers may predict the effects of biologics on volumetric BMD changes

## Introduction

Rheumatoid arthritis (RA) and ankylosing spondylitis (AS) have been associated with generalized and localized inflammatory bone resorption. Increased bone formation is also prevalent in AS [1, 2]. Osteoporosis and increased riak of fragility fractures (FF) have been observed in the majority of patients [1–3]. Tumor necrosis factor α (TNF-α) induces bone loss via the stimulation of Receptor Activator Nuclear Factor kappa B ligand (RANKL) [1, 4–6] and also the resorptive capacity of osteoclasts [7, 8]. In clinical trials, anti-TNF-α agents inhibited bone loss and radiographic progression in RA [9–11]. TNF inhibition may also affect arthritis-associated osteoporosis [3, 10, 12, 13]. The suppression of systemic inflammation by these targeted therapies is crucial for the preservation of bone in RA and AS [10, 12].

BMD is assessed by dual-energy X-ray absorptiometry (DXA). Peripheral quantitative computed tomography (QCT) of the forearm is a useful to assess trabecular and cortical bone separately. DXA and QCT determine areal and volumetric bone mineral density (BMD), respectively. Peripheral QCT may be able to determine BMD in forearms or legs [14–17]. There have been some QCT studies on the effects of corticosteroids [3, 18] and denosumab [19] on bone. Some studies also confirmed bone loss in AS by QCT [20–22].

In a previous study we compared QCT and DXA in RA patients and healthy individuals [23]. Total, trabecular and cortical BMD was lower in RA compared to controls. Anti-citrullinated protein antibody (ACPA) seropositivity was associated with lower trabecular BMD. Correlation analyses suggested that areal BMD determined by DXA may correlate with volumetric BMD measured by QCT. Moreover, trabecular osteoporosis may be associated by the underlying autoimmune-inflammatory disease, while cortical osteoporosis may rather be age-related [23].

We have recently set up a mixed cohort of RA and AS patients and reported multiple effects of anti-TNF treatment over one year on bone metabolism and areal BMD including DXA and laboratory assessments [12]. We did not find any further bone loss during anti-TNF-α therapy in association with clinical improvements in both diseases. Moreover, TNF inhibition enhanced bone formation and suppressed joint destruction [12]. We found only one study, where the effects of 3-month anti-TNF treatment on small joint erosions was investigated [24].

The aim of this study is to assess QCT BMD in biologic-treated RA and AS patients. To our best knowledge, ours is the first study to assess the effects of one-year anti-TNF therapy on bone status in RA and AS that includes QCT, DXA, disease activity and bone biomarker data.

## Patients and methods

### Patients

Forty patients with inflammatory arthritis (24 RA and 16 axial radiographic AS) selected for the initiation of anti-TNF therapy but unselected for CVD (any previous CV events) and OP (T-score < -2.5) were enrolled in the study as described before [12]. In brief, patients with active

disease were recruited prior to initiating a biological therapy. Inclusion criteria included definitive diagnosis of RA or AS; high disease activity (DAS28 > 5.1, BASDAI > 4 after at least 3 months of combined conventional DMARD therapy); clinical indication of biological therapy. Exclusion criteria included acute/recent infection, standard contraindications to anti-

TNF therapy, chronic renal or liver failure. None of the patients had known primary osteoporosis prior to the diagnosis of RA or AS. None of the patients received replacement vitamin D therapy at the time of inclusion. Patient characteristics in the full (RA+AS), RA and AS cohorts are seen in Table 1. The full cohort included 24 women and 16 men with mean age of 51.5±13.6 (range: 24-77) years, while mean age at diagnosis was 42.1±13.5 (range: 17-58) years. The mean disease duration was 8.3±7.8 (range: 1-44) years. Patients with active disease were recruited prior to initiating a biological therapy. At baseline RA patients had a mean DAS28 of 4.92±1.12, while AS patients exerted mean BASDAI of 5.66±1.33. All patients started on an anti-TNF therapy at baseline and continued the same biological treatment during one year.

**Table 1:** Patient characteristics

	RA	AS	Total
n	24	16	40
female:male	21:3	3:13	24:16
age (mean±SD)(range), years	53.2±7.4 (35-77)	42.1±10.9 (24-72)	51.5±13.6 (24-77)
age at diagnosis (mean±SD)(range), years	43.5±11.6 (29-58)	29.2±8.9 (17-44)	42.1±13.5 (17-58)
disease duration (mean±SD) (range), years	8.7±8.5 (1-44)	7.0±6.7 (1-26)	8.3±7.8 (1-44)
RF positivity, n (%)	17 (71)	-	-
ACPA positivity, n (%)	14 (58)	-	-
DAS28 (baseline) (mean±SD)	4.92±1.12	-	-
BASDAI (baseline) (mean±SD)	-	5.66±1.33	-
Treatment (ETN, CZP)	14 ETN, 10 CZP	16 ETN	30 ETN, 10 CZP
Low-dose corticosteroids (< 6 mg/day methylprednisone)	8	1	9
DXA L2-L4 osteoporosis (T-score <-2.5)	2	1	3
DXA L2-L4 osteopenia (T-score<-1)	12	2	14
DXA femoral neck osteoporosis (T-score<-2.5)	1	1	2
DXA femoral neck osteopenia (T-score<-1)	11	5	16
fragility fracture history	7	6	13

*Abbreviations*: *ACPA* anti-citrullinated protein antibody, *AS* ankylosing spondylitis, *BASDAI* Bath Ankylosing Spondylitis Disease Acivity Index, *CZP* certolizumab pegol, *DAS28* 28-joint disease activity score, *DXA* dual-energy X-ray absorptiometry, *ETN* etanercept, *RA* rheumatoid arthritis, *RF* rheumatoid factor, *SEM* standard error of mean

Clinical assessments were performed at baseline and after 12 months of therapy [12]. Among the 24 RA patients, 14 received etanercept (ETN) 50 mg/week subcutaneous (SC) and 10 received certolizumab pegol (CZP) (400 mg at 0, 2 and 4 weeks, and thereafter 200 mg every two weeks SC). Altogether 12 RA patients were treated with ETN and 8 with CZP in combination with methotrexate (MTX). The other patients received anti-TNF monotherapy. RA patients did not take DMARDs other than MTX. All 16 AS patients received ETN monotherapy 50 mg/week SC. Altogether 8 RA and 1 AS patients took low-dose (<6 mg/day) methylprednisolone (Table 1).

The study was approved by the Hungarian Scientific Research Council Ethical Committee (approval No. 14804-2/2011/EKU). Written informed consent was obtained from each patient and assessments were carried out according to the Declaration of Helsinki.

### Clinical assessment

First, detailed medical history was taken as described previously [12]. We inquired for history of fragility fractures in all patients (Table 1). Further clinical assessments including physical examination were performed at baseline and after 12 months of therapy.

### Bone densitometry assessments by QCT and DXA

In order to determine volumetric (3D) BMD Single-slice peripheral quantitative computed tomography (QCT) assessments of the ultra-distal region of the dominant forearm were performed using a Stratec XCT-2000 instrument (Stratec Medizintechnik GmbH, Pforzheim, Germany) as described before [23]. Distal sites at 4% of the radius length contain mainly trabecular bone. QCT can differentiate between cortical and trabecular bone. Total, trabecular and cortical BMD values as determined by QCT are expressed as mg/cm^3^. Setting to acquire the image were 0.59 mm voxel. Analysis was completed using XCT6.00B software (Stratec Medizintechnik GmbH, Pforzheim, Germany) with measuring mask set to radius and threshold density to 269 mg/mm^3^ to define trabecular bone.

Areal (2D) BMD (g/cm^2^) of the lumbar spine (L2-L4 vertebrae) and femoral neck (FN) was assessed by DXA (Lunar DPX-L, GE Healthcare) according to standard protocol as described before [12]. Only FN was assessed in order to match forearm QCT as both FN and the radius contains mostly cortical bone.

Both QCT and DXA assessments were performed within one week for each patient.

### Laboratory measurements and assessment of disease activity

Serum C-reactive protein (CRP; normal: ≤ 5mg/l) and IgM rheumatoid factor (RF; normal: ≤ 50 IU/ml) were measured by quantitative nephelometry (Cobas Mira Plus-Roche), using CRP and RF reagents (both Dialab). ACPA (anti-CCP) autoantibodies were detected in serum samples using a second generation Immunoscan-RA CCP2 ELISA test (Euro Diagnostica; normal: ≤ 25 IU/ml) as described before [12]. The assay was performed according to the manufacturer’s instructions. Disease activity of RA and AS was calculated as DAS28-ESR (3 variables) and BASDAI, respectively.

### Bone biomarkers

Serum calcium (Ca; Roche Diagnostics; normal: 2.1-2.6 mmol/l) and phosphate (P; Roche Diagnostics; normal: 0.8-1.45 mmol/l); parathyroid hormone (PTH; Roche Diagnostics; normal: 1.6-6.9 pmol/l); total 25-hydroxy-vitamin D (VITD; DiaSorin; normal: ≥75 nmol/l); osteocalcin (OC; Roche Diagnostics; normal: <41 μg/l), procollagen type I N-propeptide (P1NP; Roche Diagnostics; normal: <75 μg/l), C-terminal collagen telopeptide (CTX; Roche Diagnostics; normal: <0.57 μg/l), osteoprotegerin (OPG; Biomedica; median: 2.7 pmol/l); sclerostin (SOST; Biomedica; median: 24.14 pmol/l), Dickkopf-1 (DKK-1; Biomedica; median: 36 pmol/l), soluble RANKL (Ampli-sRANKL; Biomedica; median: 0.14 pmol/l) and cathepsin K (CATHK; Biomedica; median: 8.7 pmol/l) were determined by ELISA at baseline and 12 months after treatment initiation as reported previously [12].

### Statistical analysis

Statistical analysis was performed using SPSS version 22.0 (IBM) software as described before [12]. Data are expressed as the mean ± SD for continuous variables and percentages for categorical variables. The distribution of continuous variables was evaluated by Kolmogorov-Smirnov test. All bone variables showed normal distribution. Independent and paired two-tailed t-test were used to assess the differences. Nominal variables were compared between groups using the chi-squared or Fisher’s exact test, as appropriate. Correlations were determined by Pearson’s analyses. Univariable and multivariable regression analysis using the stepwise method were applied to investigate independent associations between BMD as determined by QCT (dependent variables) and other clinical, laboratory and DXA parameters (independent variables). The β standardized linear coefficients showing linear correlations between two parameters were determined. The B (+95% CI) regression coefficient indicated independent associations between dependent and independent variables during changes. General linear model (GLM) repeated measures analysis of variance (RM-ANOVA) was performed in order to determine the additional effects of multiple parameters including therapy on 12-month changes of QCT BMD. In this analysis, partial η^2^ is given as indicator of effect size, with values of 0.01 suggesting small, 0.06 medium and 0.14 large effects. The power was estimated with G-Power software. P values < 0.05 were considered significant. In order to exclude the effects of multiple comparisons (Table 2 and S1), we applied Bonferroni’s correction. We have 3 variables, therefore in these cases, p<0.017 was considered significant.

**Table 2:** Significant correlations of QCT BMD parameters with DXA BMD and laboratory parameters, as well as disease activity in the full RA+AS cohort^a^

	QTOTBMD-0	QTOTBMD-12	QTRABBMD-0	QTRABBMD-12	QCORTBMD-0	QCORTBMD-12
**Disease activity**						
*DAS28/BASDAI-0*	NS	**NS**	R=-0.402 p=0.015	NS	NS	R=-0.465 p=0.005
**DXA parameters**						
*DXL2L4BMD-0*	R=0.399 p=0.016	NS	R=0.437 p=0.008	NS	NS	R=0.493 p=0.004
*DXL2L4BMD-12*	R=0.406 p=0.014	NS	R=0.436 p=0.008	NS	NS	R=0.501 p=0.004
*DXFEMBMD-0*	NS	NS	NS	NS	NS	R=0.563 p=0.001
*DXFEMBMD-12*	NS	R=0.402 p=0.014	NS	NS	NS	R=0.547 p=0.001
**Bone biochemical markers**						
*CTX-12*	NS	NS	R=0.400 p=0.011	NS	NS	NS

^a^Bonferroni’s correction was applied in order to exclude the effects of multiple comparisons. Therefore in this case p<0.017 is considered statistically significant

*Abbreviations*: *BASDAI* Bath Ankylosing Spondylitis Disease Activity Index, *BMD* bone mineral density, *CATHK* cathepsin K, *CTX* C-terminal telopeptide, *DAS28* 28-joint disease-acitivity score, *DXFEMBMD* DXA femoral neck BMD, *DXL2L4BMD* DXA lumbar 2-4 vertebral BMD, *NS* non-significant, *OC* osteocalcin, *P1NP* procollagen type I N-propeptide, *QCORTBMD* QCT cortical bone mineral density, *QTOTBMD* QCT total bone mineral density, *QTRABBMD* QCT trabecular bone mineral density, *RANKL* Receptor Activator of Nuclear κB Ligand

## Results

### Osteoporosis and osteopenia among the patients

Among the RA patients, 2 had osteoporosis (T-score<-2.5) and 12 had osteopenia (T-score<-1) at the lumbar spine (L2-L4 vertebrae) as determined by DXA. In the femoral neck, one had osteoporosis (T-score<-2.5) and 11 had osteopenia (T-score<-1) at the femoral neck region. In the AS subset, one patient had L2-L4 and one had femoral neck osteoporosis. Two patients had L2-L4 and 5 had femoral neck osteopenia. Seven RA and 6 AS patients had fragility fracture in their history. In the full RA/AS cohort, 3 patients had L2-L4 and two patients had femoral neck osteoporosis, while 14 patients had L2-L4 and 16 had femoral neck osteopenia. Altogether 13 patients had previous fragility fracture (Table 1).

### Effects of anti-TNF therapy on volumetric BMD

**Fig. 1:**
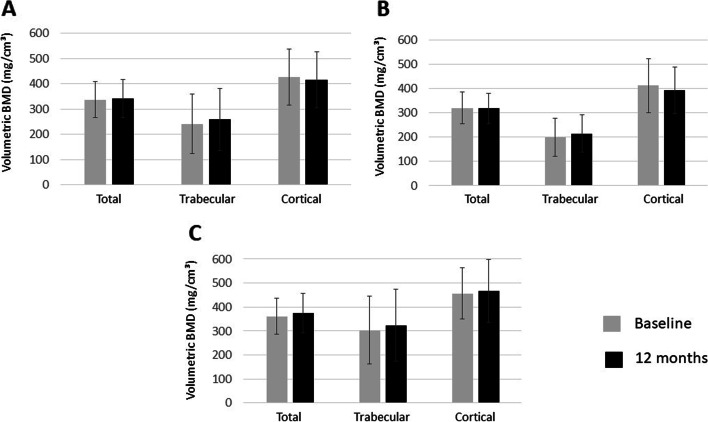
One-year changes of volumetric BMD upon TNF inhibition in the full RA+ASW cohort (**A**), as well as in the RA (**B**) and AS (**C**) subsets. Total, trabecular and cortical BMD values as determined by QCT are indicated.

In the full cohort, as well as in RA or AS patients, we did not observe any further bone loss during ETN or CZP therapy over one year as determined by peripheral QCT. In the full cohort, total volumetric BMD (QTOTBMD) (340.9±75.3 vs 336.7±71.8 mg/cm^3^; p=0.669), trabecular BMD (QTRABBMD) (258.0±123.0 vs 241.1±117.6 mg/cm^3^; p=0.283) and cortical BMD (QCORTBMD) (415.1±111.9 vs 426.3±110.1 mg/cm^3^; p=0.591) did not change significantly after 12 months of treatment versus baseline, respectively (Fig. 1). In the RA subset, QTOTBMD (319.9±66.3 vs 318.3±62.9 mg/cm^3^; p=0.828), QTRABBMD (199.4±77.2 vs 213.9±76.8 mg/cm^3^; p=0.362) and QCORTBMD (412.5±111.0 vs 391.6±95.6 mg/cm^3^; p=0.296) also did not change significantly over the one-year period (Fig. 1). Finally, similar pattern was observed in AS patients with respect to QTOTBMD (361.8±74.4 vs 374.8±81.4 mg/cm^3^; p=0.561), QTRABBMD (303.6±141.0 vs 324.1±150.0 mg/cm^3^; p=0.528) and QCORTBMD (456.3±107.1 vs 466.5±131.6 mg/cm^3^; p=0.845) (Fig. 1). As of note, QTOTBMD, QTRABBMD and QCORTBMD values were significantly higher in AS compared to RA both at baseline and after 12 months of therapy (p<0.05) (Fig. 1).

### Correlations between volumetric BMD and other clinical, laboratory and DXA parameters

Volumetric BMD determined by QCT at baseline (QTOTBMD-0, QTRABBMD-0 and QCORTBMD-0) and after 12 months of anti-TNF therapy (QTOTBMD-12, QTRABBMD-12 and QCORTBMD-12) were correlated with disease activity, lumbar and femoral neck DXA BMD and bone biomarkers described above (Table 2). In the mixed cohort of RA and AS patients, either DAS28 or BASDAI was considered as marker of disease activity, respectively. In the simple Pearson’s correlation analysis using Bonferroni’s correction, DAS28/BASDAI-0 inversely correlated with QTRABBMD-0 (p=0.015) and QCORTBMD-12 (p=0.005) (Table 2). Disease activity did not correlate with QTOTBMD and QCORTBMD at baseline (Table 2). Also, QCT outcomes did not correlate with CRP (Table 2). Similar correlations were not observed in the RA or AS subset (Table S1).

With respect to correlations between QCT and DXA, baseline or 12-month QTOTBMD, QTRABBMD and QCORTBMD values showed multiple correlations with baseline or post-treatment DXL2L4BMD and DXFEMBMD values in the full cohort (Table 2). Similar correlations were observed in RA but not in AS (Table S1). Thus, in general, volumetric BMD determined by QCT positively associated with areal BMD indicated by DXA in the full cohort and in RA (Table 2 and S1).

Regarding bone laboratory biomarkers, in the full cohort, QTRABBMD-0 positively correlated with CTX-12 (p=0.011) (Table 2). In RA, SOST-0, while in AS, RANKL-0 and RANKL-12 exerted variable correlations with QCT BMD values (Table S1). Thus, in general, bone resorption or resorption markers variably associated with volumetric BMD in the full cohort, as well as in the RA and AS subsets (Table 2 and S1).

### Independent determinants of volumetric BMD

In the full cohort, the univariable analysis showed that QTOTBMD-12 may be inversely determined by disease activity at baseline (DAS28/BASDAI-0; p=0.030). The negative determinants of QCORTBMD-12 included DAS28/BASDAI-0 (p=0.005), CATHK-0 (p=0.025) and CATHK-12 (p=0.033) (Table 3). The negative associations of QTOTBMD-12 (p=0.030) and QCORTBMD-12 (p=0.012) with baseline disease activity was also confirmed by multivariable analysis (Table 3). In RA, VITD-0 was positively associated with post-treatment QTRABBMD in both the uni- and multivariable analyses (Table S2). No such correlations were found in AS (Table S2). Thus, baseline disease activity, CATHK or VITD may be associated with the outcome of QCT BMD after one-year treatment (Table 3 and S2).

**Table 3:** Univariable and multivariable analysis of determinants of QCT parameters in the full RA+AS cohort

Dependent variable	Independent variable	Univariable analysis				Multivariable analysis			
		β	p	B	95% CI	β	p	B	95% CI
*QTOTBMD-12*	*DAS28/BASDAI-0*	-0.344	0.030	-23.704	-44.940 - -2.468	-0.344	0.030	-23.704	-44.940 - -2.468
*QCORTBMD-12*	*DAS28/BASDAI-0*	-0.465	0.005	-46.802	-78.388 - -15.215	-0.425	0.012	-40.917	-72.310 - -9.704
	*CATHK-0*	-0.408	0.025	-7.342	-13.695 - -0.990		NS		
	*CATHK-12*	-0.390	0.033	-7.504	-14.355 - -0.653		NS		

*Abbreviations*: *BASDAI* Bath Ankylosing Spondylitis Disease Activity Index, *BMD* bone mineral density, *CATHK* cathepsin K, *DAS28* 28-joint disease-acitivity score, *NS* non-significant, *QCORTBMD* QCT cortical bone minderal density, *QTOTBMD* QCT total bone mineral density

Finally, GLM RM-ANOVA was performed to assess combined determinants of volumetric BMD changes over the 12-month period. In the full cohort, the change of QTRABBMD between baseline and 12 months was determined by the anti-TNF treatment together with higher baseline VITD levels (p=0.031) (Table 4). In addition, TNF inhibition and lower CATH-K determined QCORTBMD changes over the one-year period (p=0.006) (Table 4). Similarly, in RA, baseline VITD or CATHK, while in AS, baseline RANKL may enhance the effects of treatment on one-year changes in QCT BMD (Table S3). Thus, baseline VITD, CATHK and RANKL may be important factors in the effects of treatment on QCT BMD changes (Table 4 and S3).

**Table 4:** Significant results of general linear model (GLM) repeated measures analysis of variance (RM-ANOVA) test determining the effects of treatment and other independent variables on QCT parameters as dependent variables in the full RA+AS cohort

Dependent variable	Effect	F	p	Partial η^**2**^
*QTRABBMD 0-12*	Treatment * higher VITD-0	5.006	0.031	0.116
*QCORTBMD 0-12*	Treatment * lower CATHK-0	9.000	0.006	0.243

*Abbreviations*: *CATHK* cathepsin K, *GLM* general linear model, *QCORTBMD* QCT cortical bone minderal density, *QTRABBMD* QCT trabecular bone mineral density, *RM-ANOVA* repeated measures analysis of variance, *VITD* 25-hydroxyvitamin D

## Discussion

To our best knowledge, these may be the first data on the effects of one-year anti-TNF therapy on bone status in RA and AS that includes QCT, DXA, disease activity and bone biomarker measurements. During the follow-up, there was no progression of structural bone loss at peripheral sites. The associations we found may reflect the effects of systemic inflammation of bone status in RA and AS. This is in line with the EULAR and treat-to target recommendations for RA [25] and AS [26] treatment that we would goal at lowest possible disease activity in patients to prevent structural damage at inflamed sites.

It has been established, that bone loss occurs in both RA and AS [1–3, 5, 11–13, 23]. The pathogenesis of osteoporosis involves TNF-α and anti-TNF biologics are able to block generalised bone loss in arthritides [4, 8, 10, 12, 27, 28]. However, local bone loss in the forearm, close to the wrist region, has not yet been evaluated in biologic-treated RA and AS patients. In the same cohort of RA and AS patients, no further bone loss was observed during ETN and CZP therapy over 12 months [12]. DXA determines areal BMD. Peripheral QCT of the forearm may have additive value in assessing bone status in inflammatory rheumatic diseases. QCT determines volumetric BMD and may also indicate bone status in vertebral and cortical bone compartments [17, 18, 23, 29, 30]. Feehan et al [31] developed a customized protocol in order to determine metacarpal head and shaft, as well as distal radius bone quality by QCT. Felder et al [18] applied QCT to assess the effects of corticosteroids on the bone in RA. Caparbo et al [21], as well as Devogelaer et al [22] confirmed bone loss in AS by QCT. Korkosz et al [20] evaluated the spine by QCT and could not prove any relationship between baseline new bone formation and generalized bone loss after 10 years of follow-up.

In the present study, no further bone loss was observed during one-year anti-TNF therapy in both RA and AS as determined by QCT volumetric BMD assessments. There have been very few reports on the effects of biologics on volumetric BMD. Shimizu et al [24] have recently determined 3-month changes in bone microstructure due to anti-TNF therapy. That study included 27 anti-TNF and 10 methotrexate-treated RA patients. The authors evaluated the number and volume of erosions by HR-QCT and found that TNF inhibition may slow down the progression of erosion development. Changes in the number of erosions were associated with changes in disease activity [24]. That 3-month study did not include DXA and laboratory biomarker assessments [24]. Interestingly, Yue et al [19] followed bone erosion repair upon denosumab therapy by HR-QCT.

With respect to comparison of QCT with DXA, Zhu et al [29] assessed relationship between bone density and microarchitecture by comparing hand, peripheral and axial sites by DXA of the lumbar spine and hips, as well as by QCT of the distal radius and second metacarpal head. In our recent study, total, trabecular and cortical BMD was lower in RA compared to controls. ACPA seropositivity was associated with lower trabecular BMD [23]. Here we did not find correlations between seropositivity and volumetric BMD, however, these were different RA patients all ready to receive biologics, while in our previous study, this was not the case [23]. Correlation analyses suggested that areal BMD determined by DXA may correlate with volumetric BMD measured by QCT [23]. In our study, multiple correlations were found between volumetric BMD and areal BMD determined by QCT and DXA in the full cohort and in the RA subset, respectively. Not only correlations at the same time points were observed, but baseline volumetric and areal BMD also determined areal and volumetric BMD after one-year of TNF inhibition, respectively.

Regarding disease characteristics and bone biomarkers, Aschenberg et al [32] assessed clinical, as well as catabolic and anabolic bone markers with periarticular changes measured by HR-QCT in RA. Interestingly, only disease duration but none of the bone biomarkers were associated with erosions, probably because occurrence of erosions may take some years and the follow-up time of observation is often short.

On the other hand, age and bone alkaline phosphatase correlated with anabolic changes, such as osteophytes [32]. In the present study, baseline disease activity (DAS28/BASDAI-0) inversely correlated with trabecular BMD at baseline, and with total and cortical BMD at one year at the distal radius. The uni- and multivariable analysis also confirmed that baseline disease activity independently determines total and cortical BMD after 12 months. This is in line with Shimizu et al [24] findings that showed correlations between changes in the number of erosions determined by HR-QCT and disease activity during 3-month anti-TNF therapy of RA patients. The data are exciting as in our present study, bone resorption markers CATHK and RANKL were negatively, while the bone formation markers as well as VITD were positively correlated with volumetric BMD at different time points. As our QCT measurements were performed close to the wrist, the bone biomarkers mentioned above, in combination with systemic inflammation, may play a role in the development of erosions investigated in other QCT studies [24, 33, 34].

It seems that biological therapy exerts its favourable effects on QTRABBMD changes over one year in combination with high baseline VITD levels. In other words, higher levels of VITD may enhance the beneficial effects of anti-TNF biologics on bone loss [10, 12]. Similarly, 12-months of TNF inhibition combined with low baseline CATHK levels determine QCORTBMD changes between baseline and 12 months in RA. In the same cohort we found that anti-TNF therapy decreases CATHK production in RA and AS [12]. In AS, baseline RANKL together with treatment determined one-year QCT BMD changes. We have not found any reports on the role of RANKL levels in determining outcomes in AS. In conclusion, one-year anti-TNF treatment itself, as well as disease activity, CATHK, RANKL and possibly baseline VITD are the most important denominators of volumetric BMD changes over time in RA and AS.

Our results suggest that there may be differences between RA and AS with respect to the effect of biologics on bone. Separation of the two diseases was not easy due to the relatively small patient numbers, however, some conclusions could be made. In general, volumetric BMD values whether total, trabecular or cortical were higher in AS than in RA at baseline and after treatment. Certainly, AS patients are younger but there may be other factors as well. QCT BMD values were associated with disease activity and with DXA BMD in RA but not in AS suggesting that disease activity linked to systemic inflammation may drive bone loss to a greater extent in RA compared to AS. With respect to bone markers, QCT BMD changes were associated with VITD and CATHK in RA but rather with RANKL in AS.

The strength of this study is that, for the first time, the changes in volumetric BMD assessed by QCT were evaluated in RA and AS patients undergoing anti-TNF therapy. From earlier studies, we did know that the generalised bone loss (far from joints, such as spine) can be arrested by anti-TNF agents, but we now showed that bone loss very nearly around the joints (wrists/hands) could also be arrested. We also elucidated the mechanism: one-year increase in volumetric BMD was most pronounced in patients with very low DAS28/BASDAI. Moreover, we also found that more favourable changes in QCT BMD were observed in patients with anti-TNF-induced low bone resorption markers (CATHK, RANKL) and high bone formation markers (P1NP and OC). Limitations may include the relatively low number of RA and AS patients. We did not have access to HR-QCT so we used the standard QCT technique.

In conclusion, QCT may be suitable to determine volumetric BMD in various compartments (trabecular and cortical) of the radius. QCT, as well as DXA [12] confirmed, that biologics may halt generalized bone loss. Volumetric and areal BMD values correlate with each other suggesting the value of both QCT and DXA in the assessment of bone status in arthritides. Moreover, we identified baseline parameters, such as disease activity, CATHK and possibly VITD that may predict the effects of one-year anti-TNF treatment on volumetric BMD changes over time. CATHK and VITD may be associated with changes in cortical and trabecular bone, respectively. Further, larger studies are needed to determine the value of peripheral QCT in the everyday rheumatology practice.

## Supplementary Information

**Additional file 1: Table S1.** Significant correlations of QCT BMD parameters with DXA BMD and laboratory parameters, as well as disease activity in RA and AS patients.

**Additional file 2: Table S2.** Univariable and multivariable analysis of determinants of QCT parameters in RA and AS patients.

**Additional file 3: Table S3.** Significant results of general linear model (GLM) repeated measures analysis of variance (RM-ANOVA) test determining the effects of treatment and other independent variables on QCT parameters as dependent variables in RA and AS patients.

## Data Availability

The datasets used and/or analysed during the current study available from the corresponding author on reasonable request.
