# Supplementary material for: Peripheral quantitative computed tomography in the assessment of bone mineral density in anti-TNF-treated rheumatoid arthritis and ankylosing spondylitis patients
Source: BMC Musculoskelet Disord. 2021 Sep 23;22:817. doi: 10.1186/s12891-021-04708-5 (PMC8461899; doi:10.1186/s12891-021-04708-5)
Supplement: Supplementary file 1 — Additional file 1: Table S1. Significant correlations of QCT BMD parameters with DXA BMD and laboratory parameters, as well as disease activity in RA and AS patients. [file 12891_2021_4708_MOESM1_ESM.docx]

**Table S1.** Significant correlations of QCT BMD parameters with DXA BMD and laboratory parameters, as well as disease activity in RA and AS patients

|  | **QTOTBMD-0** | **QTOTBMD-12** | **QTRABBMD-0** | **QTRABBMD-12** | | **QCORTBMD-0** | **QCORTBMD-12** |
| --- | --- | --- | --- | --- | --- | --- | --- |
| **RA PATIENTS (n=24)** | | | | | | | |
| **Disease activity** | | | | | | | |
| *CRP-12* | R=-0.493  p=0.014 | NS | NS | NS | NS | | NS |
| **DXA parameters** | | | | | | | |
| *DXFEMBMD-0* | NS | NS | NS | NS | | NS | R=0.594  p=0.002 |
| *DXFEMBMD-12* | NS | NS | NS | NS | | NS | R=0.560  p=0.004 |
| **Bone biochemical markers** | | | | | | | |
| *SOST-0* | NS | NS | R=0.560  p=0.013 | NS | | NS | NS |
|  | | | | | | | |
| **AS PATIENTS (n=16)** | | | | | | | |
| **Bone biochemical markers** | | | | | | | |
| *RANKL-0* | NS | NS | NS | NS | | R=-0.684  p=0.014 | NS |
| *RANKL-12* | NS | NS | NS | NS | | R=-0.731  p=0.007 | NS |

^*^Bonferroni’s correction was applied in order to exclude the effects of multiple comparisons. Therefore in this case p<0.017 is considered statistically significant. Abbreviations: AS, ankylosing spondylitis; BMD, bone mineral density; CRP, C-reactive protein; DKK, Dickkopf; DXFEMBMD, DXA femoral neck BMD; DXL1BMD, DXA L1 vertebral BMD; NS, non-significant; QCORTBMD, QCT cortical bone mineral density; QTOTBMD, QCT total bone mineral density; QTRABBMD, QCT trabecular bone mineral density; RA, rheumatoid arthritis; RANKL, Receptor Activator of Nuclear κB Ligand; SOST, sclerostin; VITD, 25-hydroxyvitamin D.
