# Supplementary material for: Peripheral quantitative computed tomography in the assessment of bone mineral density in anti-TNF-treated rheumatoid arthritis and ankylosing spondylitis patients
Source: BMC Musculoskelet Disord. 2021 Sep 23;22:817. doi: 10.1186/s12891-021-04708-5 (PMC8461899; doi:10.1186/s12891-021-04708-5)
Supplement: Supplementary file 2 — Additional file 2: Table S2. Univariable and multivariable analysis of determinants of QCT parameters in RA and AS patients. [file 12891_2021_4708_MOESM2_ESM.docx]

**Table S2.** Univariable and multivariable analysis of determinants of QCT parameters in RA and AS patients

| **Dependent**  **variable** | **Independent variable** | **Univariable analysis** | | | | **Multivariable analysis** | | | |
| --- | --- | --- | --- | --- | --- | --- | --- | --- | --- |
|  |  | **β** | **p** | **B** | **95% CI** | **β** | **p** | **B** | **95% CI** |
| **RA PATIENTS (n=24)** | | | | | | | | | |
| *QTOTBMD-12* | *SOST-0* | 0.462 | 0.046 | 1.099 | 0.020-2.178 |  | NS |  |  |
| *QTRABBMD-12* | *VITD-0* | 0.558 | 0.005 | 0.940 | 0.321-1.558 | 0.558 | 0.005 | 0.940 | 0.321-1.558 |
|  | | | | | | | | | |
| **AS PATIENTS (n=16)** | | | | | | | | | |
| No significant associations were found. | | | | | | | | | |

Abbreviations: AS, ankylosing spondylitis; BMD, bone mineral density; NS, non-significant; QTOTBMD, QCT total bone mineral density; QTRABBMD, QCT trabecular bone mineral density; RA, rheumatoid arthritis; SOST, sclerostin; VITD, 25-hydroxyvitamin D.

**Table S3.** Significant results of general linear model (GLM) repeated measures analysis of variance (RM-ANOVA) test determining the effects of treatment and other independent variables on QCT parameters as dependent variables in RA and AS patients

| **Dependent variable** | **Effect** | **F** | **p** | **Partial η^2^** |
| --- | --- | --- | --- | --- |
| **RA PATIENTS (n=24)** | | | | |
| *QTRABBMD 0-12* | *Treatment * VITD-0*  *Treatment * CATHK-0* | 7.921  13.102 | 0.010  0.002 | 0.265  0.435 |
| *QCORTBMD 0-12* | *Treatment * VITD-0*  *Treatment * CATHK-0* | 9.320  0.446 | 0.006  0.002 | 0.298  0.446 |
|  | | | | |
| **AS PATIENTS (n=16)** | | | | |
| *QTOTBMD 0-12* | *Treatment * RANKL-0* | 4.919 | 0.045 | 0.275 |
| *QCORTBMD 0-12* | *Treatment * RANKL-0* | 6.599 | 0.030 | 0.423 |

Abbreviations: AS, ankylosing spondylitis; CATHK, cathepsin K; GLM, general linear model; QCORTBMD, QCT cortical bone mineral density; QTOTBMD, QCT total bone mineral density; QTRABBMD, QCT trabecular bone mineral density; RA, rheumatoid arthritis; RANKL, Receptor Activator Nuclear factor kappa B; RM-ANOVA; repeated measures analysis of variance; VITD, 25-hydroxyvitamin D.
